# Am I who I say I am? Unobtrusive self-representation and personality recognition on Facebook

**DOI:** 10.1371/journal.pone.0184417

**Published:** 2017-09-19

**Authors:** Margeret Hall, Simon Caton

**Affiliations:** 1 School of Interdisciplinary Informatics, University of Nebraska at Omaha, Omaha, United States of America; 2 School of Computing, National College of Ireland, Dublin, Ireland; Dalian University of Technology, CHINA

## Abstract

Across social media platforms users (sub)consciously represent themselves in a way which is appropriate for their intended audience. This has unknown impacts on studies with unobtrusive designs based on digital (social) platforms, and studies of contemporary social phenomena in online settings. A lack of appropriate methods to identify, control for, and mitigate the effects of self-representation, the propensity to express socially responding characteristics or self-censorship in digital settings, hinders the ability of researchers to confidently interpret and generalize their findings. This article proposes applying boosted regression modelling to fill this research gap. A case study of paid Amazon Mechanical Turk workers (n = 509) is presented where workers completed psychometric surveys and provided anonymized access to their Facebook timelines. Our research finds indicators of self-representation on Facebook, facilitating suggestions for its mitigation. We validate the use of LIWC for Facebook personality studies, as well as find discrepancies with extant literature about the use of LIWC-only approaches in unobtrusive designs. Using survey data and LIWC sentiment categories as predictors, the boosted regression model classified the Five Factor personality model with an average accuracy of 74.6%. The contribution of this work is an accurate prediction of psychometric information based on short, informal text.

## Introduction

Across platforms like Facebook, LinkedIn, Twitter, and blogging services, users (sub)consciously represent themselves in a way which is appropriate for their intended audience [1–5]. However, researchers have not yet adequately addressed controlling for self-representation, the propensity to display socially responding characteristics or effects of self-censorship in online settings [2,6]; including online social network platforms. The trove of potential online social media data is vast, but the ability of researchers identifying ground truth models, and thus to verify its authenticity, is low. This can result in misleading or wrong analyses [7–10]. As such, researchers on these platforms risk working with ‘gamified,’ or socially responding personas that go beyond efforts to contain Common Method Biases (CMB) in research design [11,12]. This leaves the open question of alignment of unobtrusively gathered online data and self-reported data. In this paper, we focus on the alignment of survey methods with unobtrusive methods of gathering data from online social media.

This article has two aims:

- To explore the relationship between offline and online personalities via survey responses and self-produced text such that;
- Participant-influenced biases in publically sourced data can be mitigated.

In response to these research aims, we hypothesize that self-representation can be identified by test-based attributes (Section 2) and describe a mechanism to do so in the context of Facebook studies. For this, we employed the popular crowdwork platform Amazon Mechanical Turk, receiving survey responses and anonymous Facebook Timeline data from 509 workers (Section 3). Following on from the identification of self-representation, we discuss how it can be controlled for in broad social models (Section 4). Section 5 then discusses the implications of this work and summarizes the contribution, limitations, and points out areas for future work (Section 6).

### Conceptual background

Self-representation has been discussed in several works for online and offline fora. These studies discuss that one's tendency to truthfully disclose or censor personal information emanates from an associated intrinsic value [13–18]. Many methods including surveys, interviews, and (n)ethnographic research can identify self-representation from the first person perspective. Sentiment analysis is a promising research design for the unobtrusive identification and mitigation of self-representation bias in data at a lower overall cost [19–22]. Whilst the phenomenon of representation of self is across all social media, Facebook lends itself well for conducting such analyses as it is larger and has a higher upper bound of characters per post than its major competitor Twitter [23], and Facebook generally has set audience boundaries [24].

#### Presentation of self in online social networks

We define self-representation in accordance with Goffman [25] as controlling or guiding the impression others could make by altering the posters’ settings, appearance and manner. Goffman’s work was extended for digital fora by [3,5]. Both Hogan [3] and boyd and colleagues [5] contend self-representation is an increasingly frequent strategy in online participation and communication. In the view of Goffman and Hogan self-representation is the display of the scenario-based ideal self, rather than a pattern of deception. This view was extended by [4], who finds that self-representation online can be for expressive, communicative, or promotional purposes. However, in contrast to the work by Van Dijck [4], we define self-representation as distinct from the concept of identity contingencies [26], where self-representation is the presentation of a scenario-based idealized self and identity contingencies is the staging of a social identity marker (e.g., being a computer scientist, being from the United States) in order to highlight communal (dis)similarities. Online self-representation can be employed on social media with text typed, photos posted, emojis used, and presence/absence of group identities (among other displayed attributes).

Self-representation is also bound to time and place. In real life one must immediately respond to an interlocutor or opponent. In social networks, one has the option not to act immediately. This is even true in the case of messaging platforms using delivered/read notifications (i.e., Facebook, Whatsapp). Even these types of sites deliver notifications of messages to the front page or screen of the interface, thus allowing the user to opt to respond at a time of their choice. Local binding is functionally eliminated with online social networks [3,25]. In real life direct communication is often the social norm [27] whereas in social networks communication is more indirect. Status updates, uploading pictures, or inserting information in the "About Me" section is not directed to anyone specifically. Although one approximately knows who may be reached, it is not known who will respond [4].

Individuals self-represent due to an increase in intrinsic value [16,25]. Across studies, honesty in online representation is valued but ability and application of self-representation online has attractive socially-reinforced benefits. Qualitative interviews (n = 100) on internet dating found that the potential for self-representation is an attractive attribute of online activities [14]. A contradicting study by [13] considered an online dating environment in order to determine the extent of self-representation by users. Results of their interviews (n = 34) indicate that the users who are more ‘honest’ in self-presentation have more success in dating. Nonetheless, all interviewees noted that in their online dating profiles they attempt to reveal themselves particularly positively, and have the same impression of the profile construction of other users. [28] describe self-representation as self-monitoring, defined as the construction of a publically presented self for social interactions in their 116-person study. [28] define high self-monitors as those who carefully curate their self-presentation and low self-monitors as those who are less guarded by portraying their ‘real’ selves. They find that high self-monitors are more likely to occupy preferential positions and have higher social network density than low self-monitors, measures of the relative success of a self-representation strategy and popularity situating [29].

There is still open debate on the extent of self-representation online. For example, online self-representation was challenged by [17], who find that posters describe extensions of their actual lives in their survey and nethnography of 133 Americans and 103 Germans. In a literature review, [30] argue that self-representation is contextual. Most people use Facebook to stay in touch with people met offline, so they cannot completely detach their true identity [2,31]. Utz and colleagues established in their twinned studies of 255 and 198 Dutch participants that users shorten self-descriptions to make themselves seem more interesting. When the audience is likely to be unknown, users try to present a socially aspired self-image to be ‘popular’ [29].

#### Emotional disclosure on Facebook

Studies show that honest self-disclosure is generally more emphasized in real life and is different online [1,13,18]. [1] measured 185 then 37 participants in two studies, discovering that users communicate their positive emotions online more frequently via social posturing, finding that negative emotions in Facebook are hardly communicated. When negative (and positive) emotions are used, they tend to cluster around users groups [32,33]. The intensity of positive emotion disclosure is often linked to one’s extraversion or neuroticism levels as measured on the Five Factor personality model of [34]. Extraverts have been found to express significantly higher frequencies of positive emotions [35–37].

Facebook’s study on self-disclosure, the typing then editing, deleting, or posting of statuses and comments from 3.9 million Facebook users, found that 71% of users self-censor in some way. Males censor more than female, and Facebook posts are more frequently regulated than comments. They find that those with higher boundaries (estimated by the amount of regulations on visibility in place for a given audience member of the posting person) self-censor more, and theorize that lack of control drives self-censorship. Given that perceived lack of control is a characteristic of neurotic personalities [38–40], active self-censoring can be understood as an expression of neuroticism on social media.

#### Linguistic Inquiry and Word Count (LIWC)

This section concentrates on the properties and related finding of the text analysis package Linguistic Inquiry and Word Count (LIWC). This review is not extensive, and does not cover the multiple non-LIWC tools available to measure computational affect, psychometrics, and sentiment analysis. LIWC’s premise is that it is function and not context of the word that matters. Latent emotional and psychological states are revealed by word function more than the words actually in use. Function words comprise approximately 55% of a given language and are difficult and expensive to manipulate [41]. Function words can detect emotional states [42–45], predict psychometrics [35,46,47], as well as gender and age [48]. LIWC has been applied to predict deception [49,50], and its output has proven to outperform humans when detecting dishonest writing samples [50]. LIWC shown excellent precision and recall capacities with high but not overfitting correlations in the analysis of latent sentiment [51,52]. A number of studies discuss correlations between LIWC and personality as well as attempt prediction tasks based on the same [35,41,48,53–55]. Until now it has been found that machine learning approaches often perform better than LIWC-only approaches in prediction tasks [55,56].

Recent criticisms of LIWC’s fundamental approach suggest two problems: LIWC has yet to be thoroughly validated for different mediums of online social media data [10,30], and emerging studies report low correlation strength between existing scales or survey responses and online social media data [57]. Comparison studies by [58,59] found that LIWC and LIWC-based dictionaries (e.g., SentiStrength) had high levels of precision, word recognition, and agreement as well as good prediction accuracy [58]. In general, these studies reported that LIWC was among the top of the ranks of all tools tested for the metrics named above. This is likely due to LIWC’s focus on latent sentiment: It is more difficult to manipulate the latent emotional function and state of a word than actual word use [41,60].

#### Benchmark studies on personality and Facebook using LIWC

Two studies closely match the approach of this work and are elucidated here. The initial study applying LIWC to assess personality traits from online discourse is the work [35]. Yarkoni evaluated word usage and personality traits of 694 bloggers using LIWC 2001’s 66 categories (linguistic categories minus non-semantic categories). He employed a correlation analysis of all LIWC categories and the Five Factor Model with a False Discovery Rate criterion of 0.05. This work found strong correlations across and between LIWC and the Five Factor Model. His work reports a full feature vector of each LIWC correlation with the respective personality trait.

The work [48] also considers the interaction between personality as displayed by Facebook writing samples and LIWC. Schwartz and colleagues extracted Facebook data of 75,000 participants, analysing a corpus of 700 million words. They employed three techniques to predict the gender, age, and personality of participants. Firstly, they employed LIWC as a stand-alone tool. They compared this to the open vocabulary approach (a combination of words and n-grams) and a topics-based approach. Each technique was combined to evaluate the predictive power, using a Bonferroni correction in their evaluations. Schwartz and colleagues reported on a word and phrase basis the indicators of personality, age and gender as compared to Yarkoni, who reported LIWC categories. They report that gender can be predicted with between 78.4–91.9% accuracy. They report the explained variance but not prediction accuracy of the Five Factor Model.

This work differs on several aspects. We utilize regression modelling instead of correlations for our reporting as opposed to the Yarkoni work. However, our models are built to respect the high variable-to-predictor ratio, thus use boosted models (see Statistical Modelling for more details) which is a difference in approach to the work of Schwartz and colleagues.

We report word categories in the style of [35] rather than individual words in comparison to [48]. We argue that by following the dictionary-label approach we aid replicability of the study. LIWC’s dictionaries are curated and updated fairly regularly, meaning that words falling into these dictionaries will generally be recognized. By using only words and not classifiers, researchers run the risk of particular words or phrases falling out of usage in online language. In this case, the word-based approach would no longer be replicable.

We note that many studies exist in literature that are not analysed in depth here (see, e.g., [61–64]). These studies generally employ open language approaches [61] as opposed to our concentration on the LIWC package, or employ regression modelling without enhancements from the machine learning domain as are employed in this work [38,48,61,62].

## Materials and methods

### Personality as a tool to detect self-representation on Facebook

Given the status of the literature, an interesting question is raised on the unknown interaction between personality types, posting on Facebook, and propensity for self-representation. A link between online self-representation and real-life personality has neither been definitively addressed in cyberpsychology nor sentiment analytics literature [1,47,64] on Facebook.

Personality is good basis for the identification of self-representation due to its known relationships in on- and offline fora [48,64,65] and stability [34,53,54]. Based on the findings of [35,48,53,54,62] we assume that personality is identifiable from online social media data, and that these traits can be isolated with the LIWC package. H1 and H2 support that, and serve as the expected literature-based benchmarks. H1a/b and H2a/b consider the current literature based discussions and further hypothesize that:

H1 Self-representation is characterized by withdrawing or enhancing psychometric characteristics on Facebook.
H1a Positivity bias (enhanced positivity and withdrawn negativity) is a characteristic of self-representation on Facebook.
H1b Enhanced confidence is a characteristic of self-representation on Facebook.
H2 Personality is detectable and is not mitigated via self-representation.
H2a Online self-representation cannot distort digital traces of personality that they become undetectable.
H2b LIWC features detect the attributes of personality on Facebook.

### Research design

This study design was reviewed by the National College of Ireland’s ethics committee and approved following a full review. The data anonymized are available under https://doi.org/10.5281/zenodo.852652. To facilitate our study, 509 Amazon Mechanical Turk (AMT) workers completed psychometric surveys via a Facebook application. In use for personality is the Big Five Inventory introduced by [34], human flourishing as presented by [66] and the online social media usage survey of [67], modified to be used for Facebook. The modified mechanisms of [66,67] can be found in the Online Appendix (S1 Text. Online Appendix to: Am I Who I Say I Am?), and are represented as [SM#] and [HF#] forthwith. We recognize that many psychometrics exist that could be indicative of self-representation, but the ones in use are thoroughly researched and have strong literature-based benchmarks, and thus are the most appropriate for this analysis.

AMT has proven a reliable platform for conducting online behavioural experiments [68–71]. AMT has been found to be more representative of diversity than standard samples, and is similar to the standard Facebook population [30]. AMT has also been used in similar research designs where psychometrics and Facebook are simultaneously investigated [72].

An initial screening question based on the Instructional Manipulation Check was employed in order to minimize ‘click-through’ behaviour [30,68] in order to increase the reliability of the results. Payments of US$ 0.74 were issued at the end of the survey, equating to 1 cent per question. Regardless of users’ privacy settings allowing timeline extraction or not, all 509 workers were paid with and for survey completion. The study was launched over a 24-hour period to accommodate differences in time zones.

Participants’ data including IDs were automatically one-way hashed for user privacy, with timeline, survey, and worker payment being tied to the hashed ID. This is established as a best practice in [73]. Text-based data was automatically fed into the LIWC processing tool. A summarized privacy statement and informed consent document were presented on the entry page of the AMT HIT (Human Intelligence Task). A full privacy statement was available, detailing the uses of data and steps taken to guarantee privacy in line with [71]. At no point were identifying information available to the research team, only post-processed aggregated data [71,73,74]. After the analysis for this paper was conducted all data was destroyed to completely mitigate all possibilities of de-anonymization similar to that reported in [75] and to also ensure that the terms and conditions of the MTurk platform were not compromised.

**Fig 1:**
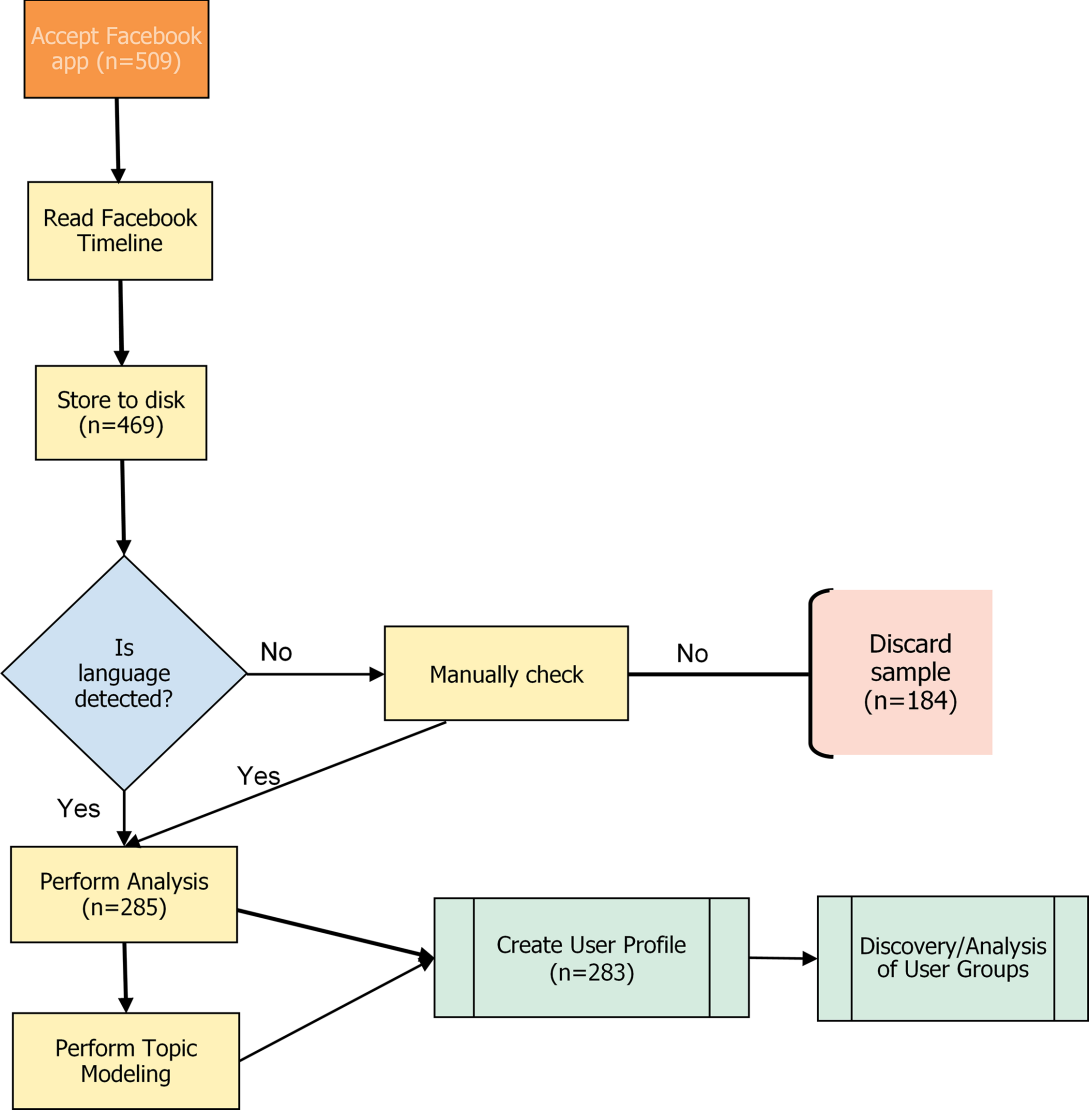
Workflow illustrating the steps to acquire, analyse, and interpret text data.

As participants completed the survey, a PHP-based Facebook application simultaneously accessed and hashed their unique Facebook ID, and via Facebook’s Open Graph API (application programming interface) accessed participants’ Facebook timelines for offline analysis (Fig 1). Workers were given an option to opt out of the HIT at the stage where it linked to their Facebook profile or abandon the HIT at any other point. Privacy-aware users were able to hide their activities from the app.

A Facebook popup screen detailed the types of data requested by the app. The app extracted only posts, i.e., status updates, participants made to their timelines. Other post types such as comments, shares, profile data and updates, etc. are excluded as they are not fully self-produced texts or could be excessively identifying. While this type of constraint can create researcher bias by potentially culling messages from the list of retrieved posts [76], we are considering the online presentation of self. Text produced by other users or the platform do not serve the same purpose. It is also an ethical grey zone to harvest the comments of participants’ friends without their direct consent [30].

### Statistical modelling

**Fig 2:**
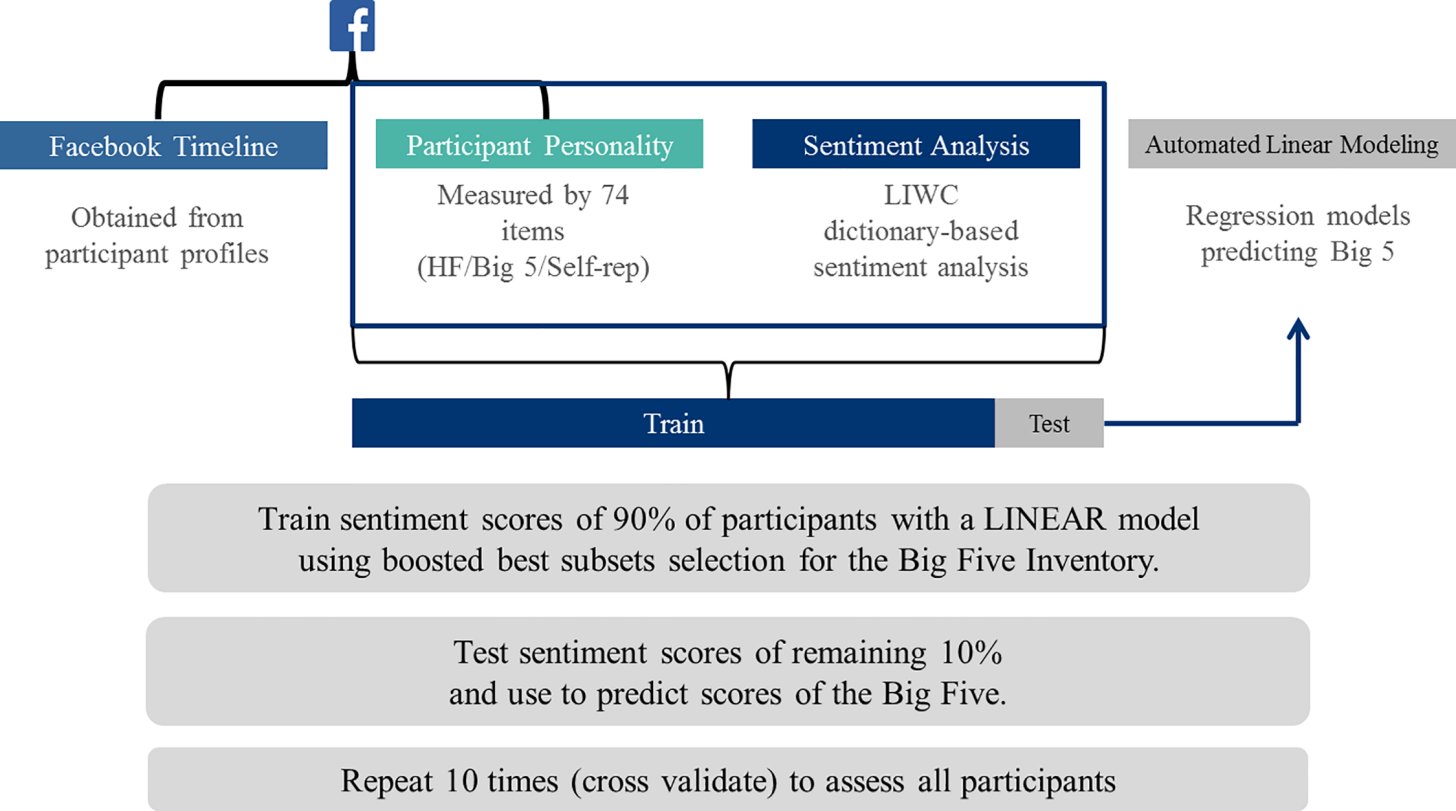
Model representation of regression analysis.

We investigate the (dis)similarity between commonly applied methods for psychometric analysis (specifically the Five Factor personality model) with a profile constructed by applying LIWC to text data sourced from the social network platform Facebook (see Fig 2). In juxtaposing these two profiles, we statistically analyse whether there are any relationships (latent or otherwise) and/or predictive capabilities in the text-based profiles. Restating the general hypothesis for this work, we expect any deviations in these profiles to be indicative of self-representation (H1). Correspondingly, as we have a psychometric inventory for each participant to hand (via the Five Factor personality model) we can statistically assess which components of our higher dimensional text-based profile account for these differences (H2b). Thus, we provide researchers with a preliminary model to redact the effects of self-representation in online platforms; specifically, Facebook.

Two statistical procedures are heavily utilized in this work, namely Spearman’s ρ and Automatic Linear Modelling (SPSS Statistics version 24). In additional, a One-Way ANOVA was performed to assess mean differences for one case and bi-nominal regression was employed in the case of discrete choice variables. While linear relationships exist in the data, some cases are non-normally distributed. [77] notes that Spearman’s ρ outperforms other correlation methods in cases of contaminated normal distributions, and is robust to Type III errors (correctly rejecting the null hypothesis for the wrong reason(s)). This justifies the use of ρ rather than Pearson’s r, in spite of the fact r tests on true values rather than ranks (thus monotonic relationships).

Automatic Linear Modelling is a machine learning extension of regression modelling and is employed for personality detection. Our analysis utilizes the boosted, best-subset model using Adjusted R^2^ as the model evaluation criteria. This is consistent with data mining approaches as suggested in [78–81]. Regression in SPSS version 24 is ruled out as it is limited to step-wise methods only, cannot conduct an all-possible subset analysis (used here for exploratory reasons), does not automatically identify and handle outliers, and cannot accommodate a model with a high variable to observation ratio [82]. Automatic linear modelling is more robust against Type I and II errors in comparison [82]. 10-fold cross validation is automatically employed by the model [82,83]. It is important to note that SPSS uses cross-validation as a part of the model building phase, therefore the individual folds have no meaning as cross-fold validation is used as the optimisation component in boosting. This is standard in boosted processes, as the weak learners are progressively compiled [83,84].

A boosted model explores iteratively learning weak classifiers with respect to a distribution by adding them to a final strong classifier [85]. When weak classifiers are added, they are typically weighted in some way that is usually related to their accuracy [86]. After a weak learner is added, the data is reweighted. This forces misclassified predictors gain weight and predictors that are classified correctly to lose weight. Thus, future weak learners focus more on the predictors that previous weak learners misclassified [78,83]. This is supported by expanding the model to a best subset approach. While computationally more intensive compared to the more common stepwise approach that economizes on computational efforts by exploring only a certain part of the model space, the all-possible-subsets approach conducts an intensive search of a much larger model space by considering all possible regression models from the pool of potential predictors [82]. This aids prediction accuracy. Pseudo-codes for the AdaBoost algorithm employed can be found in [83,84,87]. Outliers with a Cook’s Distance smaller than one were retained when they were observed to not have an undue influence on the data [88].

Boosted models are popular machine learning extensions to standard regression models, and can be employed in high-dimensional data scenarios [89]. The process of splitting the data into training and testing sets and cross-validating it tend to guard from overfitting [78]. Boosted models return strong empirical results [87] for relatively small increases in computational complexity. Most importantly, given the approach’s weight on the previous fold’s misclassified results, and assessing many weak predictors in classifying results (see above paragraph), it is expected to return highly accurate predictions [78,85,87]. As an additional step, nested 10-fold cross-validation was employed as a mechanism to evaluate the overarching model. Although Automated Linear Modelling employs cross validation in boosted model training, concerns about potentially overfitting the data can still exist. Thus, by employing nested cross-validation (cross-validating a model built using cross-validation) additional insight into the quality and performance of the resultant model is provided. The reported error estimations are less prone to overfitting and therefore are more adequate for model evaluation. This procedure additionally required SPSS Modeller (version 18) as SPSS Statistics cannot accommodate nested cross-validation.

## Results

In order to provide context, first noteworthy descriptive statistics of the data across demographic dimensions are provided, then key data cleaning and transformation processes are outlined. Subsequently, descriptives of each profile type; namely surveys and text-based via LIWC are presented and discussed, before compared with each other as well as the findings of [48]. Finally, a predictive model is proposed where key LIWC categories indicative of self-representation are discussed as a mechanism to control for self-representation. In order to provide context, first noteworthy descriptive statistics of the data across demographic dimensions are provided, key data cleaning and transformation processes are also outlined. Subsequently, descriptives of each profile type; namely surveys and text-based via LIWC are presented and discussed, before compared with each other as well as the findings of [35,48]. Finally, a predictive model is proposed where key LIWC categories indicative of self-representation are discussed as a mechanism to control for self-representation.

### Descriptive attributes of the population

Following standard online survey guidelines [90,91], participants who completed in less than nine minutes were excluded from the analysis, as well as those with unit or item non-responses (n = 40, or 7.9% of the sample population). Participants were nearly evenly split between the United States and India. The largest language group was English with 285 timelines predominately using English. 73% of participants self-reported to be aged 35 or younger. Gender of the participants is evenly split between women and men, with one non-disclosure and one choice of ‘Other.’ 37% reported being unemployed and 57% completed at least a bachelor’s degree. While this does not reflect a normalized population, a younger sample with higher educational achievements is close to the Facebook population [23].

Of the 285 English profiles, 283 have profiles with 50 or more words over the lifetime of the profiles. Sensitivity analyses indicated that the 50 word threshold was the lower limit for robust results, which is 20 words shorter than the next lowest benchmark found in IBM’s Personality Insights program with its 70-word cutoff [92]. Only the 283 English profiles with more than 50 words are used for LIWC analyses unless otherwise noted. Table 1 illustrates some descriptive categories considering the mean, standard deviation, and median of the profiles, as well as the frequency of words with more than six letters and words per sentence, all measures of linguistic maturity. The average word count per worker is 9,379, just slightly over the average of [48], at 9,333 words per participant.

**Table 1:** Average and Standard Deviation per profile (n = 283).

Per Profile	Mean	Standard Deviation	Median
Words Used	9379	1578	1726
+6 Letter Words	17	.37	16
Words/Sentence	109	50	17

### Self-reported attributes of self-representation

There are some generally interesting results dealing with self-reported contact patterns and motivation of use outside of self-representation issues revealed by the Spearman’s ρ and binomial regression analyses. Participants who use Facebook frequently also update their profiles frequently (r_*s*_(337 = .292, *p* < .005) [SM 1/2], though those with a higher number of friends have a negative relationship with the frequency of logins (r_*s*_(337 = -.314, *p* < .005) [SM 1/3]. A negative relationship also exists between number of Facebook friends and the number of updates (r_*s*_(337 = -.252, *p* < .005) [SM 2/3].

Family, and on and offline friends are major interest areas in this sample. Participants who use Facebook to show what they know and can are less interested in contacting family than all other groups (on and offline friends, unknown people) (Exp(B) = 0.5, *p* = 0.071) [SM 9H/SM4]. Those who mainly like status updates are most likely to contact family members (Exp(B) = 2.320, *p* = 0.006) [SM 1D/SM4]. Participants who use Facebook in order to be recognized by others and are half as likely to have offline friends on Facebook as the rest of the population (Exp(B) = 0.550, *p* = 0.085), and are twice as likely to be interested in contacting family members on Facebook (Exp(B) = 1,989, *p* = 0,067) [SM 9C/4]. An exception here is those who want recognition and support from other users: they are half as likely to contact family members (Exp(B) = 0.406, *p* = 0.011) [SM 9E/4]. Men are less interested in maintaining contact with family on Facebook as women (Exp(B) = 0.393, *p* = 0.001) [SM4], and those who frequently like videos are twice as likely to use Facebook for contacting their family (Exp(B) = 2.502, *p* = 0.004) [SM5/4]. Participants whose profile picture does not show their face are half as likely to want to contact offline friends and are more interested in finding unknown online friends (Exp(B) = 0.413, *p* = 0.007) [SM 11F/4], as well as participants who agree with the statement ‘I can determine myself what I do or do not show others’ (Exp(B) = 1.344, *p* = 0.033) [SM14B/4].

### Written attributes of self-representation on Facebook

**Fig 3:**
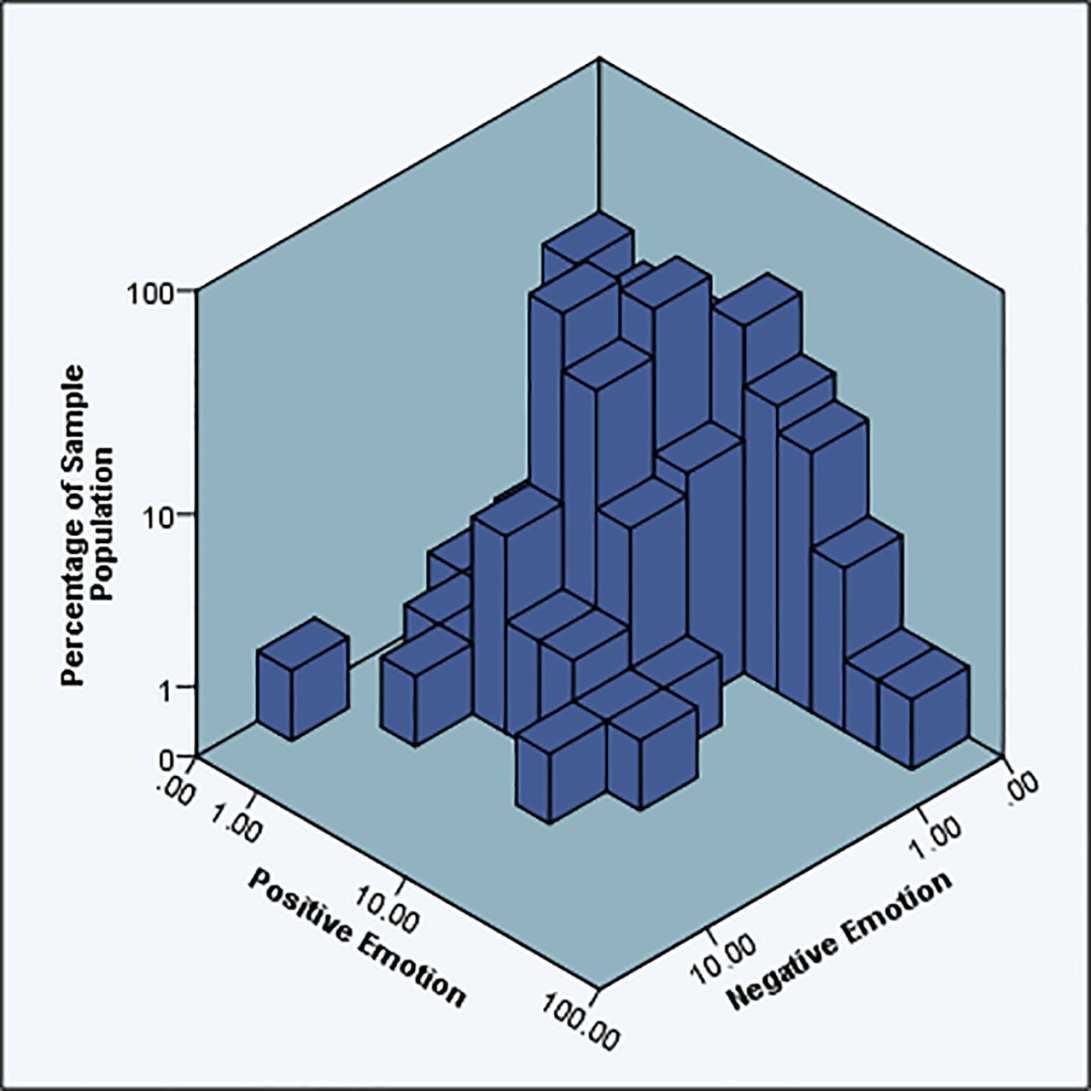
Positive and negative sentiment usage across the sample population (logarithmic scale).

As seen in Fig 3, participants generally communicate their positive emotions frequently (an average of 6.16% of each timeline), where negative emotions on Facebook are hardly communicated (2.06% of all data). This is encouraging as it is in line with LIWC standards as established by [60,93]. It is also in line with the work [1] who name this positivity bias to be social posturing. It must be noted that a contributing factor to this difference could be that LIWC has been found to generally have positive polarity in its algorithm [58]. However, 60% more words in the LIWC dictionaries are associated with negative sentiment than positive sentiment. Given that difference, it is likely that the positivity bias in this dataset is in fact a display of social posturing: people represent themselves to be more positive and less negative on their Facebook profiles, an affirmation of H1a. We note that this could also be a contributing factor to the findings of [44].

**Fig 4:**
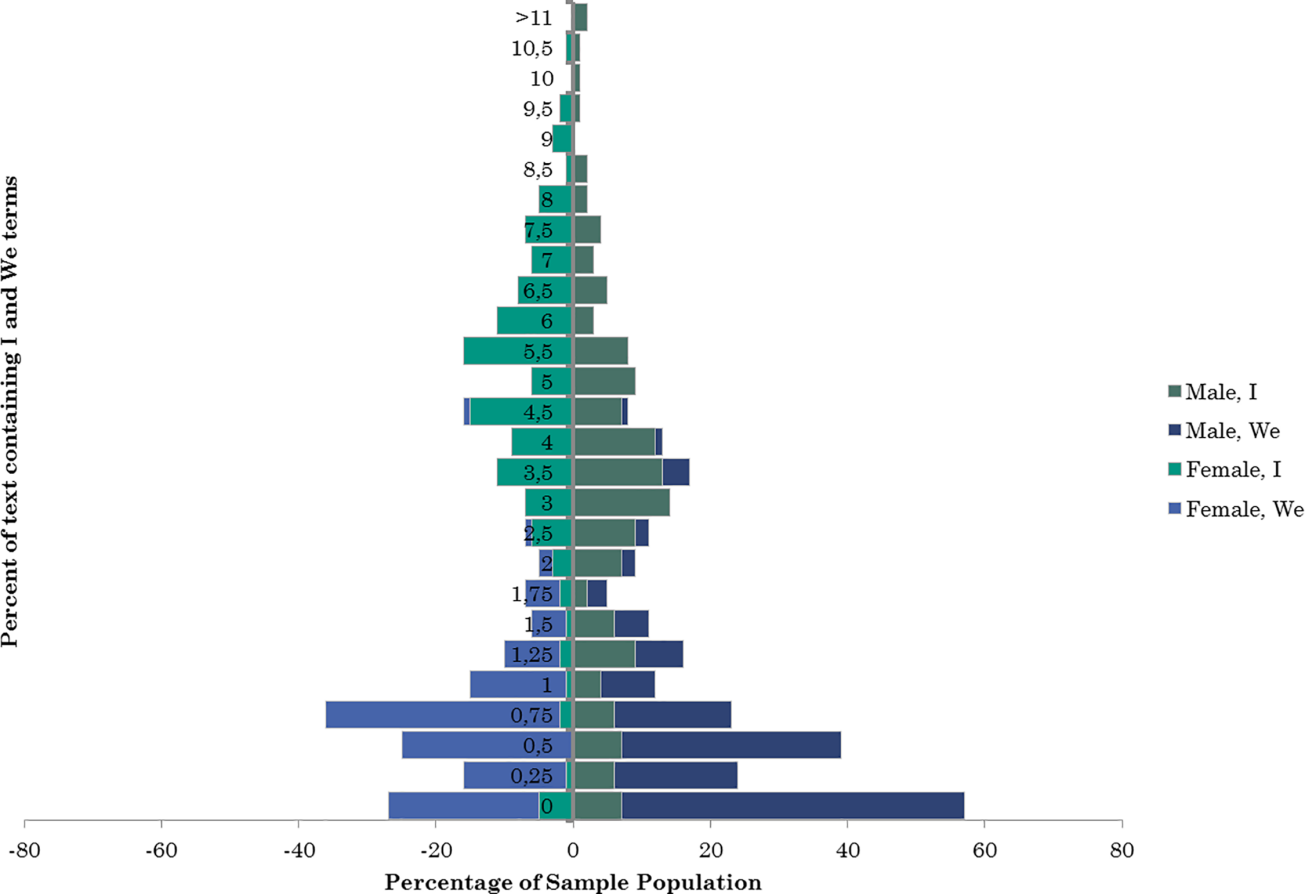
Gendered usage of confidence-expressing statements on Facebook profiles.

The analysis also looked at expressed confidence as a measure of self-representation (H1b). This is measured by the frequency in usage of first person singular and third person plural; where people that are more confident use ‘I’ words less than ‘We’ words [47,93]. We tested the demographic groups established in the survey with an ANOVA (Fig 4) and found a significant difference in gender (Gender F(2,279) = 11.893, p < .0005; Wilks' Λ = .921; partial η2 = .079). Males use more first person singular terms. Our findings cannot reject a difference between third person plural between men and women (First Person Plural (We) F(1,280) = .643, p = .423; partial η2 = .002), whereas first person singular has a significant difference in gendered usage (First Person Singular (I) F(1,280) = 23.405, p < .0005; partial η2 = .077). There was homogeneity of variance-covariance matrices, as assessed by Box's test of equality of covariance matrices (p = .002). Males are significantly more likely to present their confidence by use of ‘I’ words in their online personas. Based on the findings of [6,48], this is an unexpected and contradictory finding. This supports emerging findings that women express less confidence than men do, and thereby does not support overt self-representation specific to online social networks (H2b).

### Detecting personality from online responses and online discourse

In order to mitigate self-representation, the attributes indicating personality must first be addressed. This section discusses the predictors of the variables with the strongest predictive coefficients from the entire list of possible 136 variables (survey items and LIWC categories) and also introduces models with only data that would be available from Facebook profiles (the LIWC categories) to define the relationships between LIWC and psychometrics (Tables 2–6) (H2b). Applying the data mining technique referred to in the Methodology section (refer to Fig 2 for the model representation), we regress 136 variables of survey responses and LIWC categories on each of the five personality traits of the Five Factor model, then regress the 80 variables representing LIWC categories. It is worth noting that the same process was completed for the prediction of human flourishing. The correlations of extraversion and neuroticism to well-being are strong enough ([r_s_(282) = .357 *p* < .0005] / [r_s_(282) = -.263 *p* < .0005]) that further analyses are precluded. We introduce these attributes as personality vectors (Tables 2–6). Tables 7 and 8 display and discuss the prediction accuracy and explained variance as well as the nested cross-validation of these values of the five traits considering all 136 variables.

**Table 2:** LIWC dictionary attributes significantly predicting the trait openness.

Model Term Openness	Coefficient	Lower	Upper	Std. error	*T*	Sig
**Intercept**	3.058	2.781	3.334	0.14	21.766	0.000
**Anger**	0.108	0.006	0.209	0.052	2.086	0.038
**Abbreviation**	0.077	0.017	0.138	0.031	2.511	0.013
**Dashes**	0.035	0.005	0.066	0.015	2.316	0.021
**Recognized by Dictionary**	0.007	0.001	0.013	0.003	2.408	0.017
**Words per Sentence**	0.001	0	0.003	0.001	2.809	0.005
**Inclusion**	-0.033	-0.063	-0.002	0.015	-2.107	0.036
**Apostrophes**	-0.042	-0.083	0	0.021	-1.97	0.05
**Discrepancies**	-0.078	-0.141	-0.016	0.032	-2.462	0.014
**Humans**	-0.107	-0.19	-0.024	0.042	-2.524	0.012
**Motion**	-0.117	-0.191	-0.044	0.037	-3.131	0.002
**Semi Colon**	-0.458	-0.83	-0.086	0.189	-2.423	0.016

**Table 3:** LIWC dictionary attributes significantly predicting the trait conscientiousness.

Model Term Conscientiousness	Coefficient	Lower	Upper	Std. error	*T*	Sig
**Intercept**	3.232	3.002	3.462	0.117	27.717	0.000
**Assent**	0.449	0.156	0.741	0.149	3.021	0.003
**Home**	0.258	0.119	0.397	0.071	3.657	0.000
**Hear**	0.168	0.023	0.313	0.074	2.286	0.023
**Occupation**	0.068	0.025	0.112	0.022	3.1	0.002
**Quotation**	0.066	0.003	0.129	0.032	2.052	0.041
**Cognitive Mechanisms**	0.056	0.021	0.09	0.018	3.16	0.002
**Apostrophes**	0.044	0.001	0.086	0.022	2.001	0.046
**+6 Letters in Words**	0.013	0.004	0.021	0.004	2.865	0.005
**Recognized by Dictionary**	0.01	0.002	0.017	0.004	2.497	0.013
**Period**	-0.009	-0.016	-0.002	0.003	-2.549	0.011
**Self**	-0.027	-0.052	-0.003	0.012	-2.22	0.027
**Prepositions**	-0.028	-0.05	-0.006	0.011	-2.51	0.013
**Tentative**	-0.076	-0.128	-0.024	0.027	-2.863	0.005
**Motion**	-0.079	-0.156	-0.003	0.039	-2.043	0.042
**Communication**	-0.097	-0.163	-0.031	0.033	-2.9	0.004
**Optimism**	-0.097	-0.18	-0.014	0.042	-2.289	0.023
**School**	-0.1	-0.195	-0.004	0.048	-2.061	0.04
**Family**	-0.138	-0.266	-0.011	0.065	-2.135	0.034
**Parentheses**	-0.139	-0.274	-0.004	0.069	-2.021	0.044
**Music**	-0.176	-0.349	-0.002	0.088	-1.992	0.047
**Inhibition**	-0.335	-0.548	-0.123	0.108	-3.108	0.002

**Table 4:** LIWC dictionary attributes significantly predicting the trait extraversion.

Model Term Extroversion	Coefficient	Lower	Upper	Std. error	*T*	Sig
**Intercept**	2.97	2.638	3.301	0.168	17.63	0.000
**Semi Colon**	0.466	0.023	0.908	0.225	2.073	0.039
**Hear**	0.206	0.058	0.354	0.075	2.739	0.007
**Anger**	0.204	0.022	0.386	0.092	2.21	0.028
**Achieve**	0.172	0.105	0.239	0.034	5.052	0.000
**We**	0.172	0.063	0.281	0.055	3.12	0.002
**Abbreviations**	0.125	0.048	0.202	0.039	3.199	0.002
**Quotation**	0.101	0.031	0.17	0.035	2.865	0.005
**Colon**	0.067	0.019	0.114	0.024	2.779	0.006
**Present**	0.028	0.006	0.051	0.011	2.541	0.012
**Words per Sentence**	0.003	0.001	0.004	0.001	4.049	0.000
**Exclamations**	-0.017	-0.033	-0.001	0.008	-2.094	0.037
**Other Punctuation**	-0.054	-0.075	-0.032	0.011	-4.933	0.000
**Insight**	-0.101	-0.18	-0.022	0.04	-2.518	0.012
**Negative Emotions**	-0.105	-0.204	-0.006	0.05	-2.094	0.037
**School**	-0.111	-0.207	-0.015	0.049	-2.288	0.023
**Death**	-0.568	-1.055	-0.082	0.247	-2.3	0.022
**Grooming**	-0.592	-1.158	-0.025	0.288	-2.057	0.041

**Table 5:** LIWC dictionary attributes significantly predicting the trait agreeableness.

Model Term Agreeableness	Coefficient	Lower	Upper	Std. error	*T*	Sig
**Intercept**	2.71	2.426	2.994	0.144	18.798	0.000
**Quotation**	0.151	0.09	0.213	0.031	4.843	0.000
**Certain**	0.13	0.046	0.213	0.042	3.059	0.002
**Optimism**	0.118	0.025	0.21	0.047	2.512	0.013
**Achieve**	0.083	0.023	0.143	0.03	2.739	0.007
**Abbreviations**	0.063	0.001	0.126	0.032	2.006	0.046
**Unique Words**	0.004	0.001	0.006	0.001	3.101	0.002
**Words per Sentence**	0.002	0.001	0.003	0.001	4.672	0.000
**Period**	-0.008	-0.015	-0.001	0.004	-2.26	0.025
**Other Punctuation**	-0.025	-0.042	-0.009	0.008	-2.999	0.003
**Positive Emotion**	-0.043	-0.077	-0.009	0.017	-2.481	0.014
**School**	-0.092	-0.177	-0.008	0.043	-2.15	0.033
**Hear**	-0.14	-0.272	-0.007	0.067	-2.08	0.039

**Table 6:** LIWC dictionary attributes significantly predicting the trait neuroticism.

Model Term Neuroticism	Coefficient	Lower	Upper	Std. error	*T*	Sig
**Intercept**	3.056	2.746	3.366	0.157	19.419	0.000
**Sexual**	0.322	0.118	0.525	0.103	3.116	0.002
**Feel**	0.303	0.001	0.606	0.153	1.977	0.049
**Home**	0.217	0.063	0.37	0.078	2.784	0.006
**Achieve**	0.085	0.011	0.159	0.038	2.272	0.024
**Comma**	0.052	0.016	0.087	0.018	2.845	0.005
**Article**	-0.038	-0.071	-0.005	0.017	-2.274	0.024
**References to Others**	-0.046	-0.08	-0.012	0.017	-2.696	0.007
**Question Mark**	-0.074	-0.142	-0.006	0.034	-2.142	0.033
**Motion**	-0.13	-0.224	-0.036	0.048	-2.719	0.007

**Table 7:** Prediction accuracy, explained variance, and nested cross-validation values of the five factor personality traits compared to the accuracy and explained variance of [48].

Trait Name	Prediction Accuracy (ALM)	R^2^ (ALM)	Nested CV Mean Linear Correlation	Schwartz et al. R^2^ (LIWC only)	Schwartz et al. R^2^ (LIWC combined with topics and words)
**Openness**	65.0	0.47	0.62	0.29	0.42
**Conscientiousness**	66.7	0.43	0.68	0.29	0.35
**Extraversion**	77.9	0.56	0.70	0.27	0.38
**Agreeableness**	63.5	0.46	0.68	0.25	0.31
**Neuroticism**	70.8	0.50	0.61	0.21	0.31
**Average**	68.8	0.49	0.66	0.26	0.35

**Table 8:** Performance comparison of standard ALM results and 10-fold cross-validated (CV) ALM results.

	Min CV Linear Correlation	Mean CV Linear Correlation	Max CV Linear Correlation	ALM Results
**Openness**	0.422	0.6247	0.888	0.650
**Conscientiousness**	0.549	0.6802	0.793	0.667
**Extraversion**	0.311	0.701	0.938	0.779
**Agreeableness**	0.456	0.6754	0.803	0.635
**Neuroticism**	0.354	0.6149	0.758	0.708

### Openness

Openness has the high prediction accuracy at 65.0%, and an explained variance of 47.2%. Significant at the 0.001 level for openness are the survey categories meaning [HF 4], self-esteem [HF 9], engagement [HF3], competence [HF 1], optimism [HF 5], positive emotion [HF 6], and resilience [HF 9]; the country of origin of the worker; and the LIWC category ‘feelings.’

Table 2 illustrates the relationships between the trait and LIWC categories. Anger, Abbreviations, Dashes, Recognized by Dictionary, and Words per Sentence positively predict openness; Inclusion, Apostrophes, Discrepancies, Humans, Motion, and Semi Colons are negatively predictors.

### Conscientiousness

With a prediction accuracy of 66.7% and an R^2^ (explained variance) of 43.3%, conscientiousness is described by the largest collection of LIWC categories of all five traits (Table 3). This could be an indication of the nuance of this particular trait’s expression in online dialogue. Perhaps unsurprisingly, the strongest predictor of this trait is the LIWC category Assent.

The most relevant predictors are the LIWC categories, ‘friends’, ‘down’, and ‘fillers’; survey responses ‘a profile picture that is not obviously me’ [SM11F], number of friends [SM3], ‘I understand quickly how others perceive me’ [SM 14A], assent to ‘People should present themselves on online social networks as the same person as they are offline’ [SM 8], and using Facebook to give and get information [SM 9K], and the survey measurement resilience [HF 9] and positive relationships [HF 7].

### Extraversion

Extraversion with 77.9% accuracy and R^2^ of 56.1% is related to the survey items competence [HF 1], self-esteem [HF9], meaning [HF 4], optimism [HF 5], positive emotion [HF 6], vitality [HF 10], and resilience [HF 9]; country of origin; and the survey responses ‘I understand quickly how I am perceived by others’ [SM 14A] and managing Facebook profiles with displays of albums [SM 11G].

Interestingly, those scoring high in Extraversion have a positive usage of words displaying Anger but withdrawn usage of words conveying Negative Emotions. Extroverts also use ‘We’ words (first person plural) more than the other traits, which could be a display of withdrawn confidence as expressed online (Table 4).

### Agreeableness

Agreeableness has an accuracy of 63.5% and 46.3% explained variance indicating high reliability. Highly significant are the survey items resilience [HF 8], meaning [HF 4], self-esteem [HF 9], and competence [HF 1]; country of origin; the LIWC categories ‘friends’, ‘inhibition’, ‘feelings’, and ‘assent’; and declination of ‘I can be who or what I want on my Profile page’ [SM 14D]. Unexpectedly those scoring high on this trait reflect withdrawn usage of Positive Emotion (Table 5). They score highest of all traits in attributes capturing linguistic maturity (Unique Words, Words per Sentence).

### Neuroticism

Neuroticism has a good performance (70.8% accuracy) and reasonable R^2^ (49.9%). The most significant survey items are resilience [HF 8], self-esteem [HF 9], emotional stability [HF 2], vitality [HF 10], and optimism [HF 5]; using Facebook to spy on others [SM 9D], managing presentation of self with pictures not of them [SM 11F], using Facebook to observe other people [SM 9F], and liking videos on Facebook [SM 5]. Finally, the LIWC category ‘feelings’ is highly significant. Table 6 displays an interaction between positive usage of personal achievement but a withdrawn usage of References to Others–this could indicate that the discourse of those high in neuroticism errs towards self-centred discourse.

### Model performance considering benchmark works and implications

Worker’s self-produced text is indicative of self-representation when compared to their responses to the Five Factor model (H2). The Automated Linear Modelling approach in SPSS creates meritorious model fits averaging 68.8% reference model accuracy and 48.6% explained variance as seen in Table 7, without overt signs of data overfitting (H2a).

Considering sizeable correlations between predictor groups, the unique variance explained by each of the variables indexed by the squared semipartial correlations is low. In no case was there an instance of Cook’s Distance larger than one, so all outliers were handled within the data rather than trimmed [88]. The multivariate models are statistically significant for each personality trait (*p* < .05).

When nested cross-validation is additionally performed we see an average result of 0.67 (Table 7, Table 8). While the average of the model is nearly the same, indicating goodness of the approach, there are fluctuations found in the individual constructs (Minimum and Maximums columns, Table 8). The fluctuations in the results are assumed to be a function of the program in use, namely that when in SPSS a linear model encounters a testing instance with a value it hadn’t anticipated (e.g. an attribute value outside the range of the training data provided) SPSS generally predicts $null$. Table 8 compares the minimum, maximum and average performance of nested cross-validation across the five constructs and compares the results with those of Table 7. Per-fold results are included as Supporting Information (S1 Table. Supporting Information Per-fold performance testing).

Our models have three major differentiators with the works of [35,48]. First, we find fewer categories which are significant at the 0.05 or above level per personality trait (see Tables 2–6) as compared to [35]. We see the reduced dimensionality as a strength of our approach. It indicates that the representation of the five traits is more compact than in the benchmark works [35,48], and is likely more generalizable. Second, the strength of the coefficients in our model are considerably higher than the LIWC-only results reported in [48]. This implies that our method has competitive prediction accuracy while utilizing fewer features with stronger statistical power. Finally, an advantage of our approach (boosted, best-subset regression modelling) is the superior performance considering explained variance. The reported explained variance of the LIWC-only approach in [48] with a standard regression model reached an average of 26%, and 35% when combined with other features. Our approach averages an explained variance between 56–43%. Given this work’s near-replication of the psychometric instruments as well as known relationships between them (e.g., well-being, extroversion, and neuroticism) our reported difference is unlikely to be solely due to differences in sample size. This suggests that while other approaches (e.g., latent semantic analysis [94], open-word approaches [48,61], or correlation studies [35]) are meritorious, LIWC-only approaches when combined with machine learning extensions are also appropriate for the task. Indeed, the performance increase in comparison with standard liner models and other linguistic approaches suggests that future research should consider employing such (relatively light) machine learning approaches in the future for more accurate, reliable results.

### Implication: Personality is a tool for mitigating self-representation

Having established a compact representation of the five personality traits of [34] detected from LIWC data as it represents Facebook data, researchers can use the results reported in this work as personality vectors. Personality vectors in this case are the collated LIWC categories reported in Tables 2–6. Researchers may apply the vectors to Facebook-based data when investigating psychometrics in order to represent a more realistic view of the subject. This contributes a method for social researchers to verify psychometric baselines of subjects. Having done this, researchers are able to mitigate the effects of socially responding personas in online social media data. This delivers a closer representation to the in real personality of the subject than is currently available.

## Discussion and conclusion

The key findings of this research are that self-representation in online social media is an identifiable phenomenon, that self-representation can be isolated, and a smaller number of indicators than previously reported can be used to do so. Moreover, it opens an interesting discussion on the impact of self-representation on social media analyses, both from the perspective of the researcher validating social models, and the subject with respect to the intent of such behaviours. To our knowledge this is the first work that validates Facebook applying LIWC as a stand-alone tool for the identification of personality traits and self-representation. Similar studies have validated other text inputs (e.g., [35]), or have approach feature creation from an individual word basis [48,64]. Finally, the accuracy of our results was aided by employing a machine learning extension to the regression model (boosted regression modelling), increasing accuracy dramatically.

Self-representation was identified in a number of indicators. Positive affectivity and withdrawn negative emotions are identifiable across the workers’ profiles. Withdrawn negative affect is a particularly indicative of self-representation (H1a). However, confidence follows expected patterns across genders (H1b). Male participants appear more confident in their written profiles than females. As this is a finding in emergent literature, this cannot be understood as an overt measure of self-representation. Personality is still detectable even when self-representation is present (H2a), and LIWC-only features have meritorious performance in comparison to latent semantic methods like the open vocabulary approach of [48] (H2b). Our reported accuracies were enabled by creating a fitting model for personality prediction as opposed to off-the-shelf prediction models.

The stated aims of this research are twofold: establishing the relationship between offline and online personalities in order to mitigate such biases in publically sourced data. In accomplishing these goals, this research creates a generally applicable method for the design of cross-disciplinary methods and the analysis of social media data. Such a method is impactful in both research arenas and commercial domains, in that it allows the study designer to approximate participant baselines without highly intrusive mechanisms. A strength of this study is its consideration and application of the findings from recent cyberpsychology literature.

In a systematic manner, this research detailed the experimental design, data collection, and analysis. Common method biases are addressed and appropriately eliminated when identified. The method allows for replication by careful detailing of the steps, (pre)processing of data and models built. A major contribution is addressing method biases in the harvesting and analysis of social media data. This research utilizes the entire data stream as posted by the individual per profile, mitigating sampling errors. It also names common markers of the phenomena of self-representation based on simple LIWC categories and psychometrics that allow researchers to mitigate its effects in future research. With personality and mood validated and a sentiment analysis performed on the lifespan of a user’s Facebook timeline, we can now measure the propensity of a user to portray themselves in opposition to their truthful, psychological baseline.

We propose that researchers can apply this method of personality isolation to their analyses of publically sourced data in order to mitigate the effects of self-representation. This supports the goal of (Big) data-driven personality research being both precise and accurate. Such an approach has diverse applications in that it allows for a new personality-based estimator from which to deduce generalizations from publically accessible text onto the general population. With self-representation identified and removed, a valid measurement of psychometrics without necessitating expensive surveys or interviews is created.

### Limitations, future work

A limitation is the sample size, which disallows larger statements about linguistic subgroups; the non-English samples are too small for meaningful statistics. While larger than similar cyberpsychology studies found in the related work in terms of both participant number and volume of text, the study is still smaller than the largest Facebook studies to date [6,44,48]. Another drawback is that the results are tailored to Facebook–the findings of this study are unlikely to generalize to professional networking, microblogs, or visual media sites. A concluding remark on limitations is related to privacy. While the study obtained informed consent of its workers, the open question remains if workers truly understood the amount of information that was being given in the task.

Extensions of this research are closely linked to its limitations. Cross-platform analysis of the same user for their various public profiles would give future work a more nuanced view in the ways that social media users self-represent to different audiences. Such a work would fill research gaps in ‘best’ platform usage for information disbursement, creation, and influence, as well as impact for a given network. A network analysis of users and the resulting textured understanding of how users cluster and complement within a network would be a good area of future research. Such an approach would also support answering the questions of why social media users self-represent in the way they do, given a particular site.

## Supporting information

S1 Text Online appendix to: Am I Who I Say I Am? (DOCX)

S1 Table Supporting information per-fold performance testing. Model ID O–Openness; Model ID C—Conscientiousness; Model ID E–Extraversion; Model ID A–Agreeableness; Model ID N—Neuroticism
(PDF)
