# Supplementary material for: Am I who I say I am? Unobtrusive self-representation and personality recognition on Facebook
Source: PLoS One. 2017 Sep 19;12(9):e0184417. doi: 10.1371/journal.pone.0184417 (PMC5604947; doi:10.1371/journal.pone.0184417)
Supplement: S1 Text — (DOCX) [file pone.0184417.s001.docx]

Online Appendix to: Am I Who I Say I Am?

Facebook Usage:

SM1.       How often do you log into Facebook?

SM2.       How often do you update your profile?

SM3.       How many Facebook friends do you have?

SM4.       Who are you interested in contacting on Facebook?

SM5.       What do you find yourself frequently “Liking”?

SM6.       Do you leave your contact information (Email, phone number, address) public on Facebook?

SM7.       Which information about yourself do you have available on Facebook?

SM8.       To which degree do you agree with this statement? “People should present themselves on online social networks as the same person as they are offline.”

SM9.       With which of the following statements do you agree? (Choose all that apply) I use Facebook …

1. because contacting to others is simple
2. because I'm curious, about the kind of life of people I do not know
3. to be recognized by others
4. because I can observe people around me
5. to obtain support from others
6. because I can learn a lot about others without me having to be seen
7. to inform others what I'm doing
8. to show everyone what I know and what I can
9. because this is how people connect nowadays
10. because I can reach many people
11. to give something and, if necessary to get something back
12. to show a different side of myself

SM10.   Do other people present themselves differently in online and offline settings?

SM11.   Complete the following statement. I manage my image on Facebook with (Choose all that apply)

1. group memberships
2. personal interests
3. a profile picture that shows my face
4. likes
5. my Friend List
6. a profile picture that is not obviously me
7. Albums
8. my Cover photo

SM12.   Do you upload pictures to Facebook?

SM13.   Other people represent themselves on Facebook by ….

1. group memberships
2. personal interests
3. a profile picture that shows their face
4. likes
5. Friend List
6. a profile picture that is not obviously them
7. Albums
8. Cover photo

SM14.   To what extent do you agree with the following statements?

1. I quickly understand how I am perceived by others.
2. I can determine myself what I do or do not show others.
3. I can show personality completely.
4. I can be who or what I want on my Profile Page.
5. I can be more open online than in real life.
6. Online, I can present myself to everyone.

Human Flourishing Scale:

HF 1.*Competence*

Most days I feel a sense of accomplishment from what I do

HF 2. *Emotional stability*

(In the past week) I felt calm and peaceful

HF. 3 *Engagement*

I love learning new things

HF 4. *Meaning*

I generally feel that what I do in my life is valuable and worthwhile

HF 5.*Optimism*

I am always optimistic about my future

HF 6. *Positive emotion*

Taking all things together, how happy would you say you are?

HF 7. *Positive relationships*

There are people in my life who really care about me

HF 8. *Resilience*

When things go wrong in my life it generally takes me a long time to get back to normal.

HF 9. *Self-esteem*

In general, I feel very positive about myself

HF 10. *Vitality*

(In the past week) I had a lot of energy
