# Supplementary material for: Am I who I say I am? Unobtrusive self-representation and personality recognition on Facebook
Source: PLoS One. 2017 Sep 19;12(9):e0184417. doi: 10.1371/journal.pone.0184417 (PMC5604947; doi:10.1371/journal.pone.0184417)
Supplement: S1 Table — Model ID O–Openness; Model ID C—Conscientiousness; Model ID E–Extraversion; Model ID A–Agreeableness; Model ID N—Neuroticism (PDF) [file pone.0184417.s002.pdf]

Supporting Information Per-fold performance testing

Model ID' **O**	1	2	3	4	5	6	7	8	9	10
Minimum Error	-0.703	-0.505	-0.574	-0.565	-0.512	-0.82	-0.505	-0.432	-0.669	-1.843
Maximum Error	0.384	0.403	0.481	0.493	0.411	0.388	0.403	1.167	0.964	0.572
Mean Error	-0.11	-0.009	0.06	-0.023	-0.053	-0.068	0.053	0.144	0.108	-0.044
Mean Absolute Error	0.237	0.2	0.205	0.222	0.238	0.301	0.254	0.262	0.256	0.252
Standard Deviation	0.274	0.244	0.255	0.273	0.27	0.34	0.288	0.349	0.341	0.426
Linear Correlation	0.597	0.646	0.888	0.695	0.64	0.422	0.726	0.621	0.497	0.515
Occurrences	30	28	31	31	32	23	23	27	30	27

Model ID' **C**	1	2	3	4	5	6	7	8	9	10
Minimum Error	-0.521	-0.581	-0.375	-0.486	-0.638	-1.281	-0.6	-0.817	-1.173	-0.355
Maximum Error	1.073	0.793	0.356	0.602	0.56	0.364	0.606	0.567	0.444	0.496
Mean Error	0.084	0.123	-0.028	-0.064	-0.024	-0.111	-0.001	-0.017	-0.082	-0.019
Mean Absolute Error	0.264	0.247	0.157	0.212	0.228	0.221	0.274	0.211	0.233	0.239
Standard Deviation	0.349	0.303	0.196	0.264	0.282	0.317	0.323	0.284	0.319	0.275
Linear Correlation	0.549	0.677	0.777	0.58	0.7	0.688	0.602	0.768	0.668	0.793
Occurrences	34	25	34	27	32	27	27	31	25	20

Model ID' **E**	1	2	3	4	5	6	7	8	9	10
Minimum Error	-0.691	-0.415	-0.646	-1.171	-1.467	-0.242	-1.099	-0.476	-1.785	-0.456
Maximum Error	0.282	0.479	0.746	0.636	0.825	0.443	0.596	0.944	2.767	1.285
Mean Error	-0.087	0.081	0.1	-0.072	-0.065	0.098	-0.072	0.122	0.123	0.046
Mean Absolute Error	0.191	0.226	0.242	0.222	0.257	0.169	0.291	0.255	0.57	0.25
Standard Deviation	0.237	0.261	0.323	0.32	0.398	0.183	0.398	0.329	0.912	0.333
Linear Correlation	0.808	0.882	0.643	0.685	0.712	0.938	0.677	0.713	0.311	0.641
Occurrences	27	24	27	32	30	19	27	17	38	41

Model ID' **A**	1	2	3	4	5	6	7	8	9	10
Minimum Error	-0.276	-1.357	-0.651	-0.384	-0.354	-0.461	-0.774	-0.348	-0.957	-0.575
Maximum Error	0.344	0.543	0.294	0.424	0.544	0.638	1.146	0.429	1.177	0.603
Mean Error	0.037	-0.067	-0.005	0.06	0.084	0.017	0.015	0.023	0.116	-0.051
Mean Absolute Error	0.181	0.256	0.193	0.188	0.169	0.219	0.254	0.182	0.272	0.23
Standard Deviation	0.2	0.346	0.245	0.235	0.204	0.278	0.364	0.223	0.393	0.284
Linear Correlation	0.787	0.663	0.756	0.625	0.767	0.566	0.456	0.803	0.557	0.774
Occurrences	22	36	31	18	29	27	30	24	30	35

Model ID' **N**	1	2	3	4	5	6	7	8	9	10
Minimum Error	-0.836	-1.71	-1.102	-0.546	-0.566	-1.347	-0.546	-1.458	-0.716	-0.828
Maximum Error	0.976	0.72	1.341	1.043	0.951	0.416	0.791	1.105	0.809	0.781
Mean Error	-0.033	-0.191	0.227	0.036	0.026	-0.112	0.061	-0.105	0.014	-0.105
Mean Absolute Error	0.362	0.347	0.394	0.265	0.244	0.316	0.276	0.349	0.241	0.297
Standard Deviation	0.484	0.481	0.488	0.334	0.334	0.451	0.322	0.474	0.324	0.361
Linear Correlation	0.504	0.614	0.354	0.757	0.668	0.545	0.744	0.524	0.758	0.681
Occurrences	22	27	25	36	26	23	33	40	27	23
